# Use of anti-seizure medications in different types of autoimmune encephalitis: A narrative review

**DOI:** 10.3389/fneur.2023.1111384

**Published:** 2023-03-23

**Authors:** Jinyuan Du, Yi Guo, Qiong Zhu

**Affiliations:** ^1^Department of Neurology, Sichuan Provincial People’s Hospital, University of Electronic Science and Technology of China, Chengdu, China; ^2^Chinese Academy of Sciences Sichuan Translational Medicine Research Hospital, Chengdu, China

**Keywords:** autoimmune encephalitis, anti-seizure medications, seizure, antibodies, anti-NMDAR encephalitis, anti-LGI1 encephalitis

## Abstract

Seizures are the main manifestation of the acute phase of autoimmune encephalitis (AE). Anti-seizure medications (ASMs) play an important role in controlling seizures in AE patients, but there is currently a lack of consensus regarding the selection, application, and discontinuation of ASMs. This narrative review focuses on the use of ASMs in patients with AE driven by different antibodies. The PubMed, Embase, and MEDLINE databases were searched up until 30 October 2022 using prespecified search terms. We identified 2,580 studies; 23 retrospective studies, 2 prospective studies and 9 case reports were evaluated based on our inclusion criteria. Anti-N-methyl-D-aspartic-acid-receptor (anti-NMDAR) encephalitis is the type of AE that responds best to ASMs, and long-term or combined use of ASMs may be not required in most patients with seizures; these results apply to both adults and children. Sodium channel blockers may be the best option for seizures in anti-leucine-rich-glioma-inactivated-1 (anti-LGI1) encephalitis, but patients with anti-LGI1 encephalitis are prone to side effects when using ASMs. Cell surface antibody-mediated AE patients are more likely to use ASMs for a long period than patients with intracellular antibody-mediated AE. Clinicians can score AE patients’ clinical characteristics on a scale to identify those who may require long-or short-term use of ASMs in the early stage. This review provides some recommendations for the rational use of ASMs in encephalitis mediated by different antibodies with the aim of controlling seizures and avoiding overtreatment.

## Highlights

- Anti-NMDAR encephalitis is the type of AE that responds best to ASMs, and most adults and children with anti-NMDAR encephalitis do not require long-term treatment or combinations of ASMs.
- Sodium channel blockers may be considered the preferred drug type for seizure control in anti-LGI1 encephalitis, but patients with anti-LGI1 activity are prone to adverse reactions when using ASMs.
- Patients with intracellular antibody-mediated AE are more likely to use ASMs for a long period than patients with cell surface antibody-mediated AE.
- Clinicians can score the clinical manifestations (e.g., type of antibodies) on a combined scale to identify those who may require long-or short-term use of ASMs in the early stage of AE.
- Through a summary of the literature, we provide recommendations for the rational use of ASMs in encephalitis mediated by different antibodies to both control seizures and avoid overtreatment.

## 1. Introduction

Autoimmune encephalitis (AE) is an inflammatory disease that induces the production of immunoglobulin G (IgG) antibodies against extracellular (leucine rich glioma inactivated 1 [LGI1], contactin associated protein-like 2 [CASPR2], the N-methyl-D-aspartic acid receptor [NMDAR], the α-amino-3-hydroxy-5-methyl-4-isoxazole-propionic acid receptor [AMPAR], the gamma-aminobutyric acid B receptor [GABABR], etc.) and/or intracellular (glutamic acid decarboxylase-65 [GAD65], Ma2, anti-neuronal nuclear antibody type 1 [ANNA-1/Hu], etc.) neuronal antigens in the serum and/or cerebrospinal fluid. Seizures are the main manifestation of the acute phase of AE. Of 3,722 patients with clinical data (*n* = 118 studies), 2,601 patients presented with seizures (69.9, 95% confidence interval [CI] 68.4–71.4%). Anti-NMDAR autoimmune encephalitis (anti-NMDAR AE) is the most common type of AE (1985/3722, 53.3%). The probability of seizure is high in certain types of AE, such as anti-GABABR (91.1, 95% CI 85.3–95.2%), anti-GAD65 (83.1, 95% CI 72.9% ~ 90.7%), anti-LGI1 (75.2, 95% CI 71.7% ~ 78.6%) and anti-NMDAR AE (71.8, 95% CI 69.8% ~ 73.8%). In contrast, seizures occur in only 27.6% of cases of anti-glial fibrillary acidic protein [GFAP] (95% CI 12.7–47.2%) encephalitis (1).

Current studies indicate that more than 50% of patients with AE have had at least one epileptic seizure, and some will be diagnosed with epilepsy (2–5). The concept of “autoimmune epilepsy” was introduced at the International Autoimmune Conference in Switzerland in 2002 (6). Currently, an autoimmune aetiology is considered to be the main cause of epilepsy, according to the International League Against Epilepsy (ILAE) (7). Because epileptic seizures can occur in more than 60% of patients in the acute phase of AE, some scholars have previously referred to epileptic seizures secondary to AE as “autoimmune epilepsy,” but these seizures are usually controlled when AE subsides. Regarding the practical clinical definition of epilepsy proposed by the ILAE, acute provoked or acute symptomatic epileptic seizures at this stage cannot be considered epilepsy (8). Therefore, confusion between these two concepts may result in overtreatment with anti-seizure medications (ASMs). Moreover, in July 2020, the ILAE Autoimmunity and Inflammation Working Group published an article in Epilepsia titled “Acute symptomatic seizures secondary to autoimmune encephalitis and autoimmune-associated epilepsy: Conceptual definitions,” which defines the concepts of “acute symptomatic seizures secondary to autoimmune encephalitis” and “autoimmune-associated epilepsy,” the latter suggesting a persistent susceptibility to seizures (9). However, in practice, ASMs given in the acute phase of AE are continued after recovery from encephalitis or control of seizure symptoms; there is no consensus or guideline giving clear advice for the timing of drug discontinuation and application notes detailing which ASMs are most appropriate for different types of encephalitis caused by different antibodies. Therefore, the purpose of this review is to summarize the use of ASMs in patients with AE driven by different antibodies. The current consensus is that the use of ASMs alone is not sufficiently effective in treating seizures caused by AE and that it is necessary to use both ASMs and immunotherapy. To control seizures in patients with AE during the acute and recovery phases, ASMs can be administered either concurrently with or after immunotherapy. There is a consensus that immunopharmacological treatment is an important measure in the treatment of AE, but this is beyond the scope of this article.

## 2. Methods

For this narrative review, we searched the PubMed, Embase, and MEDLINE databases up to October 30, 2022. The searches included Medical Subject Headings (MeSH) terms and free text as follows: “autoimmune encephalitis” or “autoimmune encephalitides” or “autoimmune epilepsy” “limbic encephalitis” or “antibody-mediated autoimmune encephalitis” and “epilepsy treatment” or “antiepileptic drugs” or “antiseizure drugs” or “anticonvulsants” or “anti-seizure medications.” The literature search was conducted by two reviewers who independently evaluated the titles, the abstracts, and, when necessary, the full texts. The inclusion criteria were as follows: (1) human subjects; (2) publications in English or other languages but with English abstracts; and (3) diagnosis of autoimmune-related seizures or epilepsy based on clinical analysis and EEG criteria. The exclusion criteria were as follows: (1) no use of ASMs; (2) a lack of detailed data available on ASM use; and (3) no abstract available in English. The quantitative data are represented as percentages. Studies and results were qualitatively compared.

## 3. Results

**Figure 1:**
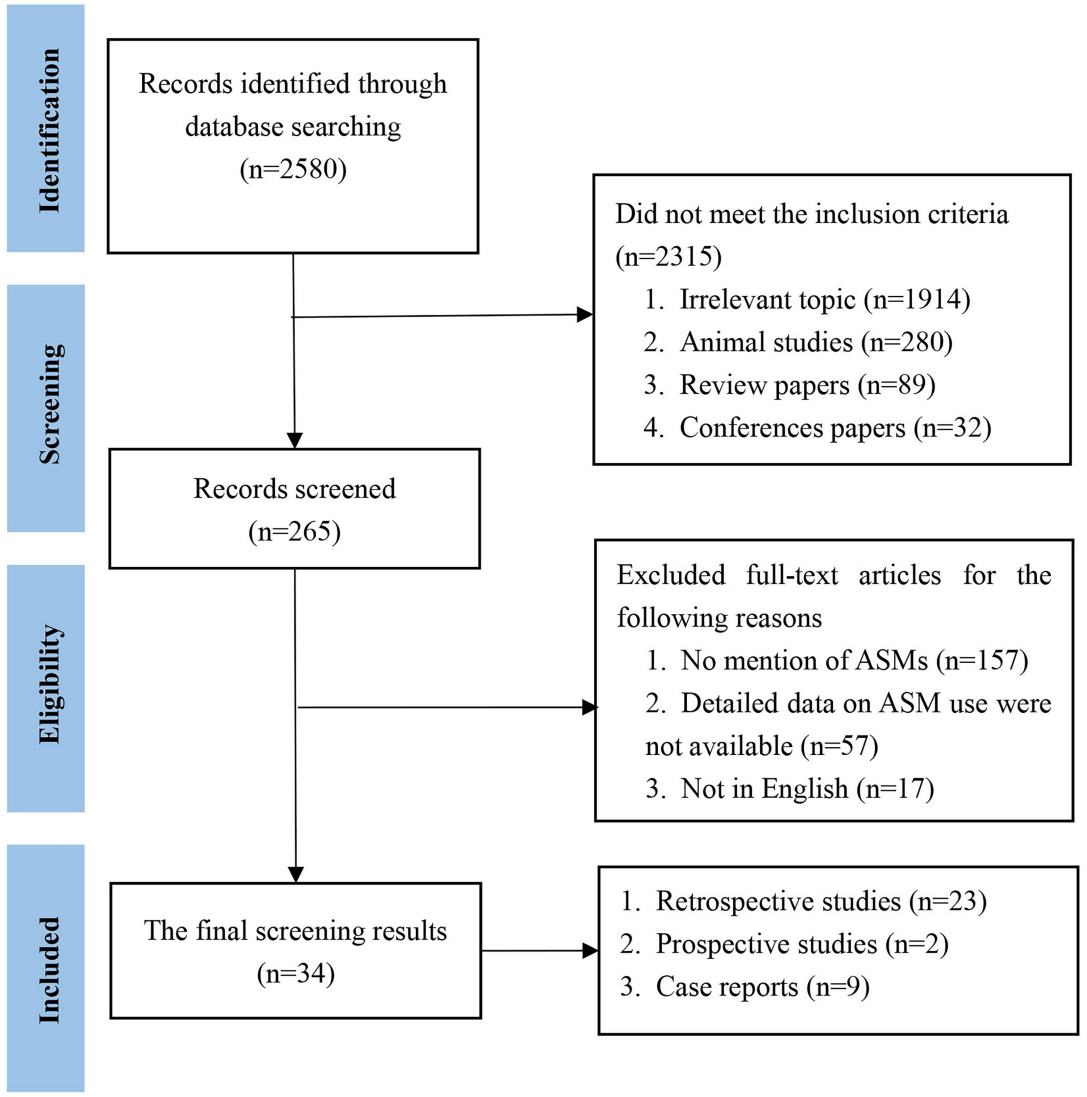
Literature search flow diagram.

We initially identified 2,580 papers. Ultimately, 34 papers met the inclusion criteria. The literature that met the inclusion criteria comprised 23 retrospective studies, 2 prospective studies and 9 case reports (Figure 1). The included cases were all diagnosed with AE and were treated with ASMs for the treatment or prevention of seizures. Cases without a confirmed diagnosis of AE or without ASM use were not included. We then classified the cases according to the type of antibody (Table 1). Among the included studies, 758 cases were reported, including 416 (55%) patients with anti-NMDAR, 168 (22%) patients with anti-LGI1, 43 (5%) patients with anti-GABABR, 10 (1%) patients with anti-VGKC, 31 (4%) patients with anti-Caspr2, 12 (2%) patients with anti-GAD65, 38 (5%) patients with anti-Ma1/2, 36 (5%) patients with anti-GFAP, and 4 (1%) patients with other antibodies.

**Table 1:** Case classification.

Different antibodies	Retrospective studies	Prospective studies	Case reports	Proportion
Anti-NMDAR	11 (*n* = 324)	1 (*n* = 88)	2 (*n* = 4)	55%
Anti-LGI1	8 (*n* = 155)	1 (*n* = 13)		22%
Anti-GABABR	5 (*n* = 42)		1 (*n* = 1)	5%
Anti-VGKC	2 (*n* = 10)			1%
Anti-Caspr2	3 (*n* = 31)			4%
Anti-GAD65	3 (*n* = 6)		2 (*n* = 6)	2%
Anti-Ma1/2	1 (*n* = 38)			5%
Anti-GFAP	1 (*n* = 35)		1 (*n* = 1)	5%
Others (mGluR5, VGCC, Hu)			3 (*n* = 4)	1%
Total	23 (*n* = 641)	2 (*n* = 101)	9 (*n* = 16)	100%

n, number of cases.

### 3.1. Use of ASMs for seizures in cell surface antibody-mediated AE

#### 3.1.1. Use of ASMs for anti-NMDAR AE

In the acute phase of anti-NMDAR AE, carbamazepine [CBZ] (*n* = 11) and oxcarbazepine [OXC] (*n* = 16) were the most commonly selected ASMs, while valproic acid [VPA] was one of the most commonly used continuous therapy agents during follow-up (10). Melissa Chavez-Castillo et al. examined 31 pediatric patients with anti-NMDAR AE and seizures. A total of 15 (48%) patients were treated with VPA; the most common ASM combination was VPA and levetiracetam [LEV]; 18 (58%) patients were treated with LEV; 13 (42%) patients were treated with phenytoin [PHT]; and 7 (23%) patients were treated with OXC (11). For seizures that remain uncontrolled after the use of one ASM in the acute phase, a combination of ASMs is needed. Haberlandt et al. reported that 15/17 (88%) patients with anti-NMDAR encephalitis took ASMs, with 3/15 (20%) patients taking 1 ASM and 12/15 (80%) patients taking multiple ASMs; 6/12 (50%) of patients taking multiple ASMs (50%) developed resistance, 1/3 (17%) of patients taking 1 ASM developed resistance, and 3 patients on multiple ASMs developed epilepsy (12). Zhong R et al. reported that among 30 patients with anti-NMDAR encephalitis, 15 (50%) were taking 1 ASM, 7 (23.3%) were taking 2 ASMs, and 8 (26.7%) were taking >2 ASMs; they found that for autoimmune encephalitis, including anti-NMDAR encephalitis, the use of more ASMs was independently associated with an increased risk of epilepsy after the acute phase of AE (13). Chavez-Castillo M et al. reported that an average of 2 ASMs were required for seizure control in 30 patients given ASMs during the acute phase of anti-NMDAR encephalitis, and more ASMs were required for patients with drug resistance, with a mean of 2.5 ASMs (11). Liu X et al. reported that in 88 patients with acute seizures in anti-NMDAR encephalitis, for 14 patients treated with a single drug (LEV, valproate, or topiramate), the median time to seizure cessation was 2 weeks; 50 patients (56.8%) received more than one ASM treatment, and the median time from ASM treatment to seizure cessation was 5 weeks (14). The data from 198 patients are shown in Table 2.

**Table 2:** Number and type of ASMs in anti-NMDAR AE.

Author	Patients using ASMs	ASMs	Number of ASMs	Therapy-resistant
Huang Q (10)	*N* = 34	*N* = 11 (32%): CBZ	*N* = 23 (68%): 1 kind	
		*N* = 16 (47%): OXC	*N* = 8 (24%): 2 kinds	
		Others: TPM, CZP, LTG, LEV, VPA	*N* = 3 (9%): >2 kinds	
Chavez-Castillo M (11)	*N* = 31	*N* = 15 (48%): VPA	An average of 2 kinds (1–5); More resistant: an average of 2.5 kinds	
		*N* = 18 (58%): LEV		
		*N* = 13 (42%): PHT		
		*N* = 7 (23%): OXC		
		Others: CBZ, CZP, LCS, LTG, TPM		
Haberlandt E (12)	*N* = 15	*N* = 10 (67%): LEV	*N* = 3 (20%): 1 kind	*N* = 1 (17%): 1 kind
		*N* = 7 (47%): VPA	*N* = 12 (80%): >1 kinds	*N* = 6 (50%): >1 kinds
		*N* = 4 (27%): CLB		
		Others: CBZ, OXC, CZP, LCS, ESM, LZP, LTG, MDZ, PB, PHT, TPM		
Zhong R (13)	*N* = 30		*N* = 15 (50%): 1 kind	
			*N* = 7 (23%): 2 kinds	
			*N* = 8 (27%): >2kinds	
Liu X (14)	*N* = 88		*N* = 14 (16%): 1 kind	
			*N* = 50 (57%): >1 kinds	

N, number; CBZ, carbamazepine; OXC, oxcarbazepine; TPM, topiramate; CZP, clonazepam; LTG, lamotrigine; LEV, levetiracetam; VPA, valproic acid; PHT, phenytoin; LCS, lacosamide; CLB, clobazam; ESM, ethosuximide; LZP, lorazepam; MDZ, midazolam; PB, phenobarbital.

When SE occurs in the acute phase of anti-NMDAR AE, treatment with combinations of multiple anticonvulsants, including phenytoin (PHT), LEV, sodium valproate (VPA), topiramate (TPM), ethosuximide, lacosamide, and clobazam, failed to achieve seizure control without propofol and midazolam (MDZ) infusions (15). For refractory SE, high doses of VPA, MDZ, isoproterenol, thiopental sodium, lidocaine, and phenobarbital (PB) may be used. High-dose TPM combined with intravenous high-dose PB or high-dose lidocaine minimizes side effects (15). Liu X et al. reported that 22 patients with SE required ASM and anesthetics, of whom 13 patients (59.1%) had controlled SE, while another 9 patients (40.9%) had unremitting or remitting SE after reduction or discontinuation of anesthetics, relapsed and eventually died in the acute phase (14). Finné Lenoir X et al. reported a case of anti-NMDAR encephalitis in a 17-year-old Asian male in whom a large number of ASMs were ineffective in his prolonged persistent epilepsy (16). In addition to conventional ASMs, Santoro et al. reported ketamine as an effective adjunctive therapy for hyper refractory persistent epilepsy in patients with anti-NMDAR receptor encephalitis, suggesting that administration of a loading dose followed by maintenance infusion resulted in clinical and/or electrogram seizure cessation in less than 48 h (17). Yen HK et al. reported anti-NMDAR encephalitis SE in 6 patients (85.7%) treated with sodium channel blockers and LEV, of which 3 patients (42.9%) received a combination of sodium channel blockers, LEV and sodium valproate, and the duration of SE in patients with combined sodium valproate and PHT at the end of SE was 2.5 ± 0.7 days (*n* = 2). Both patients had SE within significant control within 1 day of PHT use; in contrast, the duration of SE in patients who did not receive the combination of PHT, LEV and sodium valproate was 4.0 ± 4.7 days (*n* = 5) (18). Data from these studies are shown in Table 3.

**Table 3:** Use of ASMs in SE or refractory SE in anti-NMDAR AE.

Author	Patients with SE	Treatment	Outcome
Liu X (14)	*N* = 22	ASMs+anesthetics (MDZ, propofol)	*N* = 13 (59%): good response
Finné Lenoir X (16)	*N* = 1	A large number of ASMs	Poor response
Santoro JD (18)	*N* = 3	ASMs+ketamine	Good response
Yen HK (18)	*N* = 7	*N* = 3 (43%): Sodium channel blockers+LEV	Good response
		*N* = 3 (43%): Sodium channel blockers+LEV+VPA	

N, number; MDZ, midazolam; LEV, levetiracetam; VPA, valproic acid; Good response, seizure-free during follow-up; Poor response, no seizure-free and no significant improvement during follow-up.

After the acute phase of anti-NMDAR AE, it is not typically necessary to take ASMs for long periods. Early studies have found that ASMs can be gradually reduced during follow-up and that most patients who discontinue the drug within 2 years achieved seizure freedom (19). Furthermore, Zeng W et al. found that most anti-NMDAR AE patients adhered to ASMs for 3–12 months (median 0.5 years), with a mean duration of less than 1 year on ASMs and a low relapse rate (20). Qi Huang et al. reported that only 5.8% of 34 patients with anti-NMDAR AE who discontinued ASMs experienced recurrence at the 1-year follow-up, 14 patients with anti-NMDAR AE were free of seizures for at least 1 year after the last use of ASMs, and only 2 of 23 patients who discontinued ASMs within 3 months experienced recurrence (10). Yao L et al. reported that most of the 60 patients with anti-NMDAR AE discontinued ASM within 1 year, and 49 patients achieved seizure freedom at the final follow-up; however, the study included multiple antibody encephalitis at the same time, and the number of patients with NMDAR encephalitis discontinuation within 1 year was not clearly marked (21). Zhang M et al. found that at the last follow-up (range: 6–39 months), 12 of 14 patients (85.7%) with seizures achieved seizure freedom, and 10 of these 12 patients (83.3%) discontinued ASM within 1 year (22). Another study with a median follow-up of 2 years found that 33 patients (38.3%) on ASM discontinued their medication after 3 months of follow-up, 53 (61.7%) continued on ASM medication after 3 months of follow-up; additionally, no seizures were observed in patients with >2 years of follow-up, short-term (<3 months) ASM treatment was associated with long-term (>3 months) ASM treatment, and there was no difference in seizure risk factors between these groups (14). In another similar study, analysis of NMDAR brain patients with seizures in the acute phase and those who continued to have seizures after the acute phase found that the mean duration of treatment was 3.58 ± 1.08 months (range 1–6 months) for the short-term seizure group (seizures occurred only in the acute phase) and 8.40 ± 1.14 months for the persistent seizure group (seizures continued to occur after the acute phase). 1.14 months (range 7–12 months), and both groups were seizure-free after the discontinuation of ASM (23). Zhang JZ et al. reported that among 34 (68.0%) patients with anti-NMDAR encephalitis who developed seizures, 3 patients had herpes simplex virus encephalitis secondary to anti-NMDAR encephalitis and had persistent seizures; among the other patients, the seizure duration ranged from 1 ~ 47 days, and 16 patients (47.1%) did not continue ASM after discharge (24). The data from 273 patients are shown in Table 4.

**Table 4:** Withdrawal of ASMs in anti-NMDAR AE.

Author	Patients with seizures using ASMs	Follow-up	Withdrawal of ASMs	Seizure-free
Yao L (21)	*N* = 60	>2 Y	Most within 1 Y	*N* = 49 (83%) at the final follow-up
Zhang M (22)	*N* = 14 (children)	Median 20 M (6–39 M)	*N* = 10 (71%): within 1 Y, seizure remission at the acute stage;	*N* = 12 (86%) at the final follow-up
			*N* = 2 (14%): with ongoing epilepsy were treated with ASMs at the final follow-up	
Liu X (14)	*N* = 86	Median 2 Y (6–60 M)	*N* = 33 (38%): within 3 M;	100% within 2 Y; >80% within 6 M
			*N* = 53 (62%): >3 M	
Qu XP (23)	*N* = 45 (children)	>3 M	N = 34 who had seizures in 3 M: 3.58 ± 1.08 M (1–6 M);	100% at the final follow-up
			*N* = 5 who had seizures even after 3 M: 8.40 ± 1.14 M (7–12 M)	
Zhang J (24)	*N* = 34 (children)	1–6 Y (2.91 ± 1.21 Y)	*N* = 16 (47.1%): after discharge	*N* = 31
Huang Q (10)	*N* = 34	15–62 M	*N* = 23 (68%): within 3 M;	19/21 (90%)
			*N* = 11 (32%): >3 M	

N, number; M, month; Y, year.

According to studies investigating a total of 198 patients with seizures in the acute phase of anti-NMDAR AE (Table 2), valproic acid (VPA), levetiracetam (LEV), carbamazepine (CBZ), and oxcarbazepine (OXC) are the most commonly used ASMs to control these seizures. These studies in Table 2 show that the use of 1–3 ASMs is sufficient, as combining ASMs predicts an increased likelihood of developing epilepsy. VPA, LEV, and PHT can be considered in SE that occurs in the acute phase of anti-NMDAR AE; furthermore, a combination of sodium channel blockers and VPA may be an option worth revisiting for faster seizure control, and adjuvant anesthetics such as MDZ, propofol, and ketamine may be used for patients with refractory SE. These statistics in Table 4 suggest that the optimal duration of ASM use is 3 months for those with short-term seizures or a low frequency of seizures after the acute phase, while patients who still have seizures after the acute phase should slowly stop the drug after 6–12 months, and patients with persistent seizures can use ASM for a longer time.

#### 3.1.2. Use of ASMs in anti-LGI1 autoimmune encephalitis (anti-LGI1 AE)

ASMs are an adjuvant immunotherapy in the treatment of anti-LGI1 AE seizures. In one study, patients with anti-LGI1 AE presented with three main types of seizure: faciobrachial dystonic seizure (FBDS) (44%), medial temporal lobe epilepsy (MTLE)-like seizures (66%), and focal to bilateral tonic–clonic seizures (FBTCS) (77%); they showed only slight improvement after administration of ASMs, whereas immunotherapy was more effective (25). In that study, 4 (44%) patients received corticosteroids combined with intravenous immunoglobulin (IVIG), 4 (44%) patients received corticosteroids, 1 (11%) patient did not receive immunotherapy, and 8 (88%) patients received ASMs for seizures; those who received immunotherapy were significantly healthier and more likely to be free of seizures (25). LGI1 mutation-associated autosomal dominant temporal lobe epilepsy is relatively benign, and standard ASM treatment usually controls seizures (26). Van Sonderen A et al. reported 21 patients with anti-LGI1 AE who received immunotherapy in combination with ASMs over a follow-up period of more than 2 years, with a median follow-up period of 42 months. In the last year of follow-up, 28% of patients were still taking ASMs, and only 14% had seizures in the last year of follow-up (27). Huang Q et al. reported that a 72-year-old man with anti-LGI1 AE who presented with frequent seizures at presentation discontinued ASMs after 3 months, and no recurrence was reported at the 1-year follow-up (10). de Bruijn M et al. reported that for patients with anti-LGI1 AE, the odds of achieving freedom from seizures were higher with immunotherapy than with ASMs alone (19). The study by Feyissa AM et al. also concluded that immunotherapy is the treatment that best achieves freedom from seizures in anti-LGI1 AE (28). Data for these patients are shown in Table 5.

**Table 5:** Withdrawal of ASMs in anti-LGI1 AE.

Author	Patients using ASMs	Follow-up	Withdrawal of ASMs	Outcome
Li Y (25)	*N* = 8	10–45 M		Good response
van Sonderen A (27)	*N* = 21	Median 42 M	*N* = 15 (71%): within 2 Y	Good response
Huang Q (10)	*N* = 1	>1 Y	Within 3 M	Good response

N, number; M, month; Y, year; Good response, seizure-free during follow-up.

FBDS is the most specific type of attack in anti-LGI1 AE, occurring in approximately half of patients (25). Most studies have concluded that corticosteroids can control FBDS more effectively than ASMs (29). FBDS did not respond well to ASMs in a study by Gao L et al. (30). de Bruijn M et al. reported that focal seizures in patients with anti-LGI1 activity responded relatively well to CBZ, whereas FBDS showed little response to any ASM (19). In patients with FBDS, immunotherapy was associated with higher odds of achieving seizure freedom than ASM treatment (20/30 vs. 3/34, *p* = 0.001) (28). Some studies have shown positive outcomes for patients with FBDS receiving both immunotherapy and ASMs (31–33). The data from 63 patients are shown in Table 6.

**Table 6:** ASM treatment of FBDS in anti-LGI1 AE.

Author	Patients with FBDS	Therapy	Outcome
de Bruijn MAAM (19)	*N* = 25	Immunotherapy, ASM	Only ASM: poor response
Irani SR (31)	*N* = 22	ASM (2.6 kinds on average), immunotherapy	Only ASM: 19% good response Immunotherapy: 96% good response
Li LH (32)	*N* = 13	Immunotherapy+ASM (OXC, VPA, LEV, TPM)	Good response
Yu J (33)	*N* = 3	Immunotherapy+ASM	Good response

N, number; OXC, oxcarbazepine; VPA, valproic acid; LEV, levetiracetam; TPM, topiramate; Good response, seizure-free during follow-up; Poor response, no seizure-free and no significant improvement during follow-up.

Furthermore, de Bruijn M et al. reported 53 patients with anti-LGI1 AE and recommended the use of ASMs with sodium channel blocking properties (e.g., CBZ or OXC) first in the symptomatic treatment of patients with seizures. CBZ was more effective than LEV in reducing seizure frequency (*p* = 0.031) and appeared to have the best effect in reducing the frequency of focal seizures, but it tends to cause frequent rashes (19). Irani SR et al. reported that a high percentage (50%) of patients among 9 people with anti-LGI1 activity demonstrated skin reactions caused by the use of ASMs (2 PHT, 2 CBZ, and 1 VPA) (34). Shin YW et al. also reported that 10 of 20 anti-LGI1 AE patients (50%) had their ASMs changed due to adverse cutaneous drug reactions. Eight of them presented with maculopapular eruption, one presented with drug rash with eosinophilia and systemic symptoms syndrome, and one presented with eczema. Causative agents mostly consisted of aromatic ASMs. OXC was discontinued in 2 additional patients due to hyponatremia. Six patients (30%) discontinued their dose of levetiracetam because of psychiatric manifestations, including irritability/aggressive behavior (4 patients), insomnia (1 patient), and depressive mood (1 patient) (35). While the side effects of VPA typically include memory loss and tremors, the side effects of LEV consist of rash and severe behavioral changes, including severe psychotic behavior and suicidal thoughts (19).

According to the reports above, ASMs have a role in the treatment of anti-LGI1 AE seizures, but FBDS with anti-LGI1 AE responds poorly to ASMs. Overall, immunotherapy should take precedence, and ASMs are typically used for adjuvant therapy. Moreover, anti-LGI1 AE patients are prone to skin reactions and hyponatremia with CBZ and OXC or drug side effects such as adverse psychiatric reactions, memory loss, and tremor with VPA and LEV.

#### 3.1.3. Use of ASMs in other cell surface antibody-mediated AE

Huang Q et al. reported three patients with anti-GABABR autoimmune encephalitis (anti-GABABR AE), one of whom was a 35-year-old female who presented with frequent seizures at presentation and discontinued ASM after 6 months with no reported recurrence during a 1-year follow-up, while two other elderly patients (50 and 64 years old, respectively) presented with intractable seizures (10). Zhong R et al. reported on 14 patients with anti-GABABR AE, of whom 10 (71.4%) developed SE and 6 (42.9%) developed epilepsy after the acute phase of encephalitis, a much higher proportion than in anti-NMDAR AE or anti-LGI1 AE (13). It was also found that patients with anti-GABABR AE were more likely to have acute symptomatic seizures than patients with anti-NMDAR AE and that the occurrence of SE was more frequent in anti-GABABR AE (19). Yao et al. reported that patients with anti-NMDAR AE, anti-LGI1 AE, and anti-Caspr2 encephalitis all achieved effective seizure control within 2 years after concurrent immunotherapy and ASMs, whereas only 55% of patients with anti-GABABR AE had effective seizure control at the last follow-up visit after 2 years (21). Zhu F et al. reported 14 patients with anti-GABABR encephalitis: 5 patients received hormonal shock therapy, 3 patients received gamma globulin therapy, and 6 patients received hormonal shock therapy and gamma globulin. All 14 patients were on ASM, which resulted in a reduction in the number of seizures and varying degrees of improvement in both psychobehavioural disturbances and cognitive decline, but seizure freedom could not be achieved with ASM alone (36). Hainsworth JB et al. reported a case of anti-GABABR encephalitis with refractory SE that occurred with multiple ASMs and antiepileptic narcotics; after treatment with methylprednisolone, intravenous immunoglobulin, plasma exchange and rituximab, i.e., after the end of the rituximab course, the patient stopped the narcotics, had no seizure recurrence, showed significant progressive neurological improvement and was able to perform all activities of daily living independently with a normal EEG 6 months after the visit; subsequently, mycophenolate mofetil as a maintenance immunosuppressant and the antiepileptic regimen was slowly reduced to LEV monotherapy (37). Seizures in patients with anti-GABABR AE can be partially alleviated by immunotherapy combined with ASMs (10, 13, 21, 36, 37). Data for the other 40 patients are shown in Table 7.

**Table 7:** Use of ASMs in anti-GABABR AE, anti-VGKC AE, anti-Caspr2 AE and other cell surface antibody-mediated AE.

Author	Antibody	Patients	Therapy	ASM	Outcome
Yao L (21)	GABABR	*N* = 10	Immunotherapy+ASMs		*N* = 6 (60%): good response
Huang Q (10)	GABABR	*N* = 1	Immunotherapy+ASMs	ASM wean: 6 M	Good response
Zhu F (36)	GABABR	*N* = 14	Immunotherapy+ASMs		Only ASM: poor response ASM + Immunotherapy: Good response
Zhong R (13)	GABABR	*N* = 14	Immunotherapy+ASMs	*N* = 7 (50%): 1 kind *N* = 5 (36%): 2 kinds *N* = 2 (14%): >2 kinds	*N* = 10 (71%): SE *N* = 6 (43%): Epilepsy after the acute phase of encephalitis
Hainsworth JB (37)	GABABR	*N* = 1 (Refractory SE)	ASMs+narcotics+Immunotherapy	LEV, PHT, LCS, TPM, Divalproic acid	Only ASM + narcotics: poor response ASM + narcotics+Immunotherapy: good response
Pondrelli F (38)	VGKC	*N* = 2	Immunotherapy+ASM	LEV, PHT, VPA, PB, CBZ, Gabapentin	Good response
Dubey D (39)	VGKC	*N* = 8	Immunotherapy+ASM	2 kinds on average	*N* = 5 (63%): good response
Garrido Sanabria ER (40)	Caspr2	*N* = 15	Immunotherapy+ASM	LEV, Sodium channel blockers	>50% good response
Pondrelli F (38)	Caspr2	*N* = 2	Immunotherapy+ASM	LEV, VPA, LTG	*N* = 2 (100%): good response
Lancaster E (41)	mGluR5	*N* = 2	Tumor therapy ± immunotherapy ± ASM	MDZ	Good response
Finkel L (42)	VGCC	*N* = 1	Immunotherapy + ASM	Fos-PHT and TPM	Good response

N, number; M, month; LEV, levetiracetam; PHT, phenytoin; LCS, lacosamide; TPM, topiramate; VPA, valproic acid; PB, phenobarbital; CBZ, carbamazepine; LTG, lamotrigine; MDZ, midazolam; Fos-PHT, Fos-phenytoin; Good response, seizure-free during follow-up; Poor response, no seizure-free and no significant improvement during follow-up.

Pondrelli F et al. reported 2 cases of anti-VGKC autoimmune encephalitis (anti-VGKC AE), 1 with steroid immunotherapy supplemented with ASM (LEV) with improved outcome, and the other with steroid and imidazolothioprine immunotherapy supplemented with ASM, achieving no seizures (38). Dubey D et al. reported 8 cases of seizure-resistant anti-VGKC AE, most of which used immunotherapy supplemented with a median of 2 ASM, resulting in a 50% reduction in seizures after treatment in 5 (62.5%) cases (39). Anti-Caspr2 AE is a commonly used immunotherapy and ASM (38, 40). The common ASM used is LEV, and other ASMs include sodium channel blockers, VPA and LTG. The treatment results were good, and most patients achieved seizure-free status. Other cell surface antibodies that mediate encephalitis include antibodies against metabotropic glutamate receptor 5 [mGluR5] and voltage-gated calcium channel [VGCC]. They were all treated with immunotherapy and ASMs, and anti-mGluR5 encephalitis was also treated with antitumour therapy. ASMs included MDZ, Fos-PHT and TPM, and the response was good (41, 42). Data for these patients are shown in Table 7.

Based on follow-up observations of seizures in anti-GABABR AE, immunotherapy combined with ASMs was found to be effective in some patients. Patients with anti-GABABR AE are more likely to have acute symptomatic seizures and are more likely to have SE than patients with other common forms of AE, leading to an increased likelihood of long-term ASM use. The duration of ASM administration should be determined by considering all factors, and multimodal immunosuppressive therapy may be considered to improve seizures in patients who develop refractory SE. Seizures in patients with anti-VGKC AE or anti-Caspr2 AE can be mostly alleviated by immunotherapy combined with ASMs. Anti-VGKC AE is not considered definite AE but rather probable or possible antibody-negative AE. The search for VGKC-complex Ab is no longer advised, and patients should be tested for Ab against specific targets (CASPR2 and LGI1) instead (43–45).

The number of studies on other cell surface antibody-mediated AEs is currently low.

### 3.2. Use of ASMs for seizures in intracellular antibody-mediated AE

Dubey D et al. also reported 4 cases of anti-GAD encephalitis, most of which were treated with immunotherapy supplemented with a median of 1 ASM, which resulted in a 50% reduction in seizures after treatment. The number of individuals with a 50% reduction in epilepsy was 2 (50%) (39). Pondrelli F et al. reported a case of anti-GAD65 encephalitis without immunotherapy and using only ASM. The patient did not show improvement and had a poor prognosis that manifested as drug-resistant epilepsy and progressive cognitive impairment (38). Ilyas-Feldmann M et al. found that patients with neuronal surface antibodies had a significantly higher 1-year terminal seizure remission rate than patients with GAD antibodies; at the 6-year follow-up, only 1 of 5 patients (20%) using immunotherapy and ASM achieved seizure-free status (46). Jaafar F et al. reported a case of anti-GAD encephalitis that developed into super-refractory SE and was unresponsive to a combination of conventional ASMs; intravenous immunoglobulin (IVIG), high doses of corticosteroids and plasma replacement therapy were partially effective, but golimumab and ketogenic diet showed significant improvement in clinical symptoms. After 1 month of follow-up, patients on three ASMs and on a monthly golimumab were asymptomatic except for occasional sudden onset seizures (47). Mäkelä KM et al. reported a case of anti-GAD65 antibody encephalitis in a 7-year-old child presenting with super-refractory status epilepticus (SRSE). The child was given intravenous (IV) loading doses of multiple ASMs, but seizures continued to occur. Thereafter, within 48 h of the start of KD, the child achieved sufficient ketosis, and seizure frequency was greatly reduced (48). In brief, immunotherapy supplemented with ASMs, narcotics plus a ketogenic diet, or tumor therapy controls seizures in only some patients with anti-GAD65 AE and anti-Ma AE (49). If refractory SE develops, ASMs are often ineffective, while golimumab and a ketogenic diet are more effective against anti-GAD65 AE. Antibody (GAD65 and hu)-mediated AE seizures are often poorly responsive to ASMs alone (50) and only moderately responsive to immunotherapy supplemented with ASMs, narcotics plus a ketogenic diet, or tumor therapy. In anti-GFAP AE with seizures, immunotherapy supplemented with ASMs may provide a good prognosis (51, 52). Due to the good responses to treatment both were administered to children (51); however, these data are not comparable. Data for these studies are shown in Table 8.

**Table 8:** Use of ASMs in anti-GAD65 AE and other intracellular antibody-mediated AE.

Author	Antibody	Patients	Therapy	ASM	Outcome
Pondrelli F (38)	GAD65	*N* = 1	Only ASM	OXC, LEV, LTG, Perampanel	Drug-resistant epilepsy
Ilyas-Feldmann M (50)	GAD65	*N* = 5	Immunotherapy+ASM	LEV, VPA, LTG	*N* = 1 (20%): seizure free after 62 months Others: poor response
Jaafar F (47)	GAD65	*N* = 1 (superrefractory SE)	Immunotherapy (TCZ) + ASM + narcotics+ketogenic diet	LEV, LCS, PHT, CZP, VPA, MDZ, PB, Perampanel, ketamine	Poor response to Immunotherapy+ASM + narcotics Good response to TCZ + ASM + narcotics+ketogenic diet
Mäkelä KM (48)	GAD65	*N* = 1 (superrefractory SE)	Immunotherapy+ASM + narcotics+ketogenic diet	LEV, VPA, PHT, LCS, CLB, OXC, MDZ	Poor response to Immunotherapy+ASM + narcotics Good response to Immunotherapy+ASM + narcotics+ketogenic diet
Dubey D (39)	GAD65	*N* = 4	Immunotherapy+ASM	1 kind on average	*N* = 2 (50%): good response
Porta-Etessam J (50)	ANNA-1 (hu)	*N* = 1		PHT, CBZ, VPA	Poor response
Dalmau J (49)	Ma1/2	*N* = 38 (12 had seizures)	Immunotherapy and tumor therapy, ASM		Moderate response (33%)
Zhang J (52)	GFAP (+NMDAR)	*N* = 1	immunotherapy+ASM	VPA	Moderate response
Fang H (51)	GFAP	*N* = 35 (15 had seizures)	Immunotherapy+ASM	OXC	Good response

N, number; TCZ, tocilizumab; OXC, oxcarbazepine; LEV, levetiracetam; LTG, lamotrigine; VPA, valproic acid; LCS, lacosamide; PHT, phenytoin; CZP, clonazepam; MDZ, midazolam; PB, phenobarbital; CLB, clobazam; CBZ, carbamazepine; Good response, seizure-free during follow-up; Poor response, no seizure-free and no significant improvement during follow-up; Moderate response, no seizure-free but having improvement during follow-up.

### 3.3. Predictors and assessment method in different types of AE patients at increased risk for epilepsy

Zhong R et al. reported that in patients with AE (including 37 cases of anti-NMDAR AE), patients with abnormal electroencephalography (EEG) findings were 3.919 times more likely to develop epilepsy than patients with normal EEG findings, and patients with delayed immunotherapy had a 6.432-fold increase in their risk; these patients required long-term use of ASMs (13). Zhang JZ et al. reported that only patients with neocortical lesions (3 patients) continued to have seizures 1 year after discharge from the hospital; thus, patients with anti-NMDAR AE who had lesions in the limbic system and neocortex were more likely to take ASMs in the long term than those without lesions on cranial magnetic resonance imaging (MRI), suggesting that neocortical involvement is especially likely to cause epilepsy (24). In a retrospective cohort study of 39 AE patients with seizures (53), patients with surface antibodies reached first seizure-free and terminal seizure-free status more frequently, and seizure-free status was achieved after additional immunotherapy, which was not always accompanied by increased ASM doses. Seizures with surface antibodies should mostly be considered acute symptomatic and transient and not indicative of epilepsy. The maximum ASM-defined daily doses were higher in the groups with intracellular antibodies, which indicated the relative refractoriness of seizures with intracellular antibodies.

The modified Rankin Scale (mRS) score was used to assess whether 31 patients with anti-NMDAR AE needed sustained use of ASMs (11). During acute attacks, 23 patients (74%) had a maximum modified Rankin Scale (mRS) score of 5, 3 patients (10%) had an mRS score of 4, and 5 patients (16%) had an mRS score of 3. All patients showed improvements in their mRS scores at the 1-and 2-year follow-ups after the initial diagnosis. At the one-year follow-up, 15 of 29 patients (52%) had an mRS score of 0, 7 patients (24%) had an mRS score of 1, 3 patients (10%) had an mRS score of 2, and 4 patients (14%) had a poor prognosis (mRS score: 3–5). The investigators concluded that continued use of ASMs after the acute phase may be considered for SE, inadequate response to immunotherapy at 4 weeks, and high mRS scores during discharge and follow-up. In one retrospective observational cohort study, using the Antibody Prevalence in Epilepsy and Encephalopathy (APE2) and Response to Immunotherapy in Epilepsy and Encephalopathy (RITE2) scores as well as mRS scores (54, 55), Matricardi S et al. identified that cell surface antibody-mediated AE patients who received early immunotherapy may experience reductions in AE from acute symptomatic seizures to chronic epilepsy. In contrast, the severity of seizures at onset, a high number of ASMs, a poor response to immunotherapy during the acute phase and persistent interictal epileptiform discharges at follow-up may predict a worse outcome (55).

Therefore, long-term use of ASMs should be considered for patients who still have recurring seizures after the acute phase or persisting interictal epileptiform discharges at follow-up and who may develop autoimmune-related epilepsy with abnormal EEG findings, high mRS scores at discharge and follow-up, severe seizures at onset, and delayed immunotherapy or whose MRI suggests neocortical involvement. It may be safe to recommend dose reduction and eventual discontinuation of ASMs in patients with low mRS scores, early immunotherapy, good response to immunotherapy and cell surface antibody-mediated AE.

## 4. Discussion

It is clear that seizures in patients with AE should initially be treated with immunotherapy, but it is equally important to use proper ASMs to manage seizures. The effect of ASMs in the symptomatic treatment of seizures in these patients is antibody dependent; however, the evidence supporting this opinion is all based on observational studies. In this review, observational data still provide some important information on the choice, efficacy, adverse effects, and discontinuation of ASMs in encephalitis mediated by different antibodies.

Anti-NMDAR encephalitis is the most studied and recognized form of AE. In the acute phase of anti-NMDAR AE, clinicians may choose the most commonly used ASMs (VPA, LEV, CBZ or OXC) to control these seizures. For patients with drug resistance, the use of 1–3 ASMs is sufficient, as combining ASMs predicts an increased likelihood of developing epilepsy. Moreover, this conclusion is consistent with a recent large cohort study (55). VPA, LEV, and PHT may be considered for anti-NMDAR AE with SE that occurs in the acute phase. Sodium channel blockers combined with VPA may be an option worth revisiting for faster SE control, and adjuvant anesthetics such as MDZ, propofol, and ketamine may be used for patients with refractory SE. Current studies consider that patients with anti-NMDAR AE tend to have a good outcome that includes seizure discontinuation, that neither adults nor children with anti-NMDAR AE require long-term use of ASMs, and that ASMs can be discontinued as early as possible (10, 19–22, 24). Moreover, these results indicated that the optimal duration of ASM use is 3 months for those with short-term seizures or a low frequency of seizures after the acute phase, while patients who still have seizures after the acute phase should slowly stop the drug after 6–12 months, and patients with persistent seizures can use ASM for a longer time.

Patients with FBDS in anti-LGI1 AE respond poorly to ASMs alone, and immunotherapy should take precedence. Some studies have also suggested that FBDS is responsive to ASMs alone. This may be because there are subtle differences in FBDS, such as different aetiologies and symptoms in patients, which may account for the differences in the efficacy of ASMs (33). Sodium channel blockers may be preferred for treating seizures in anti-LGI1 AE, but anti-LGI1 AE patients are prone to skin reactions and hyponatremia with CBZ and OXC or drug side effects such as adverse psychiatric reactions, memory loss, and tremor with VPA and LEV. Therefore, clinicians should be cautious when introducing these medications in anti-LGI1 AE. With CBZ for anti-LGI1 AE, the cause of allergy may be related to specific pro-immunogenic human leukocyte antigen (HLA) types and increased CBZ use (56, 57); further study is needed to elucidate the exact causes of these adverse effects. For seizures in anti-GABABR AE, immunotherapy combined with ASMs was effective in most patients. Some patients are more likely to have acute symptomatic seizures and are more likely to have SE than patients with other common forms of AE, leading to an increased likelihood of long-term ASM use. Seizures in patients with anti-mGluR5, VGCC, and anti-Caspr2 AEs can be mostly alleviated by immunotherapy combined with ASMs. However, the number of studies on these cell surface antibody-mediated AEs is too small to yield reliable results.

The number of patients with intracellular antibody-mediated AE and seizures in this review was relatively small, and the most common AE was anti-GAD65 AE. Unlike cell surface antibody-mediated AE, intracellular antibody (GAD65, hu and Ma1/2)-mediated AE seizures are often poorly responsive to ASMs and, at best, only moderately responsive to immunotherapy supplemented with ASMs, narcotics plus a ketogenic diet, or tumor therapy. Consistent with these findings, a review article reported that anti-GAD65 AE exhibits only moderate responses to steroids and IVIG/plasma exchange (58). In anti-GAD65 AE with refractory SE, multimodal immunosuppressive and ketogenic-diet therapy may provide a good prognosis. Research has also suggested that immunotherapy does not stop seizures in patients with anti-GAD65 AE (59). Moreover, during assessment of predictors for AE patients at increased risk for epilepsy, studies found that intracellular antibodies may be risk factors for persistent seizures after the acute phase (53, 55, 58).

In addition to the detection of antineuronal surface antibodies, early immunotherapy was also an independent predictor of good outcome of AE. However, severe seizures at onset, a high number of ASMs, a poor response to immunotherapy during the acute phase and persistent interictal epileptiform discharges at follow-up are risk factors for the development of AE (57). Research has also shown that 2 of 163 patients with anti-NMDAR AE who developed autoimmune-associated epilepsy at ≥24 months of follow-up required continuous ASM treatment despite aggressive immunotherapy (60). Patients with AE who are at increased risk for seizures after the acute seizure phase should undergo long-term treatment with ASMs (61). Furthermore, researchers (27) identified three predictors of the duration of reasonable ASM use in patients with SE due to acute encephalitis: the admission Status Epilepticus Severity Score (STESS), seizure-free status 1 month after SE and seizure-free status 3 months after the acute phase. In their report, STESS at admission identified 37% of patients as free of seizures after the acute phase of encephalitis, the second predictor identified 44% of patients as free of seizures 1 month after the acute phase, and the third predictor identified 87% of patients as free of seizures 3 months after the acute phase. Thus, following an active phase, clinicians can score patient clinical characteristics (antibodies, immunotherapy, abnormal EEG, neocortical lesions, severity of seizures at onset, a high number of ASMs, recurring seizures after acute phase) on scales (APE2, RITE2, STESS, and mRS scores) to identify those who may require long-or short-term use of ASMs in the early stage.

The current research on AE mainly focuses on diagnosis and immunotherapy, and there are no randomized controlled trials on the use of ASMs in these patients. This review included relatively few cases from observational studies. Thus, interpretation of the results in terms of comprehensiveness and credibility should include consideration of the limitations. More clinical studies are needed to obtain further evidence.

## 5. Conclusion

In conclusion, seizures in patients with cell surface antibody-mediated AE, especially anti-NMDAR AE, are often responsive to immunotherapy supplemented with ASMs and most adults and children with anti-NMDAR AE do not require long-term treatment with ASMs. To evaluate the withdrawal time of ASMs in different types of AE patients, combining clinical manifestations with a scale should be considered in clinical practice. Further research is needed to standardize and inform the rational use of ASMs as well as to identify the mechanism of seizure in AE mediated by different antibodies.

## Author contributions

All authors contributed to the conception, design of the study, and interpretation of the data. JD and QZ were responsible for data and literature acquisition. JD wrote the first draft. All authors have provided final approval of the version of the manuscript submitted for publication.

## Funding

This work was supported by the National Natural Science Foundation of China (No. 81401081), the Scientific Research Foundation of Sichuan Provincial People’s Hospital for Doctors or Youths (No. 30305030589), and Natural Science Foundation of Sichuan (Grant No. 2022NSFSC1545).

## Conflict of interest

The authors declare that the research was conducted in the absence of any commercial or financial relationships that could be construed as a potential conflict of interest.

## Publisher’s note

All claims expressed in this article are solely those of the authors and do not necessarily represent those of their affiliated organizations, or those of the publisher, the editors and the reviewers. Any product that may be evaluated in this article, or claim that may be made by its manufacturer, is not guaranteed or endorsed by the publisher.
